# Hepatitis-C-Related Hepatocellular Carcinoma, Still a Relevant Etiology beyond a Hepatitis C Infection Cure

**DOI:** 10.3390/cancers16081521

**Published:** 2024-04-16

**Authors:** Elena Vargas-Accarino, Mónica Higuera, María Buti, Beatriz Mínguez

**Affiliations:** 1 Liver Diseases Research Group, Vall d’Hebron Research Institute (VHIR), Vall d’Hebron Barcelona Hospital Campus, 08035 Barcelona, Spain; elena.vargas@vhir.org (E.V.-A.); monica.higuera@vhir.org (M.H.); beatriz.minguez@vallhebron.cat (B.M.); 2 Department of Medicine, UAB Campus, Universitat Autònoma de Barcelona (UAB), 08193 Cerdanyola del Vallès, Spain; 3 Centro de Investigación Biomédica en Red de Enfermedades Hepáticas y Digestivas (CIBERehd), Instituto de Salud Carlos III, 28029 Madrid, Spain; 4 Liver Unit, Vall d’Hebron Hospital, Vall d’Hebron Barcelona Hospital Campus, 08035 Barcelona, Spain

**Keywords:** hepatocellular carcinoma, hepatitis C, viral hepatitis, HCV

## Abstract

**Simple Summary:**

This study investigates the etiological agents of de novo hepatocellular carcinoma (HCC) in a recent period, considering the evolving landscape of viral hepatitis prevention, therapeutic advancements, and the rising challenges of alcohol consumption and obesity. Analyzing 352 patients diagnosed with de novo HCC, the primary underlying causes were alcohol-related liver disease (33.3%) and hepatitis C (30.7%). Metabolic-dysfunction-associated steatotic liver disease (12.2%), mixed ALD and hepatitis C (8.2%), and chronic hepatitis B (6%) were also identified. Variations were observed in patient demographics, BCLC stage, and cirrhosis prevalence concerning HCC etiology. Despite improved antiviral therapy accessibility, HCV and alcoholic liver disease remain dominant contributors to HCC.

**Abstract:**

Background: In the past decades, global changes, including hepatitis B vaccination, hepatitis B and C antiviral therapies, and the increasing prevalence of steatotic liver disease, have influenced the landscape of liver cancer etiologies. Methods: We performed a retrospective study focused on the etiological factors of de novo hepatocellular carcinoma (HCC) diagnoses in an academic center between 2019 and 2022. Results: Among 352 consecutive patients with HCC, alcohol-related liver disease was the predominant etiology (33.3%), followed by hepatitis C (HCV) infection (30.7%). Significant associations were found between HCC etiology and patient demographics, BCLC stage at diagnosis, and cirrhosis prevalence. Conclusions: Whereas accessibility to antiviral therapy is granted, HCV infection remains as one of the main HCC etiologies. MASLD-related HCC, although growing globally, is not as relevant in our area. Strong public policies need to be implemented to prevent alcohol consumption, the main etiology of liver disease and liver cancer.

## 1. Introduction

Hepatocellular carcinoma (HCC) is the fourth most common cause of cancer-related deaths and the sixth in terms of incident cases, with 900,000 new cases and more than 800,000 deaths occurring in 2020 [1,2]. HCC remains a global health challenge, and its incidence is growing worldwide [3]. It has been estimated that new liver cancer cases will increase by 55% between 2020 and 2040, with a possible 1.4 million individuals being diagnosed in 2040 [4].

The majority of HCC cases occur in patients with underlying liver disease, mostly cirrhosis, and well-known related risk factors, such as chronic viral hepatitis infection (hepatitis B virus (HBV) or hepatitis C virus (HCV)), alcohol-related liver disease (ALD) and metabolic-dysfunction-associated steatotic liver disease (MASLD) [3]. Other less frequent risk factors for HCC include primary biliary cholangitis, haemochromatosis, and α1-antitrypsin deficiency, among others [5].

In the past decades and still in some countries, the main underlying cause of HCC was chronic HBV and HCV infection and alcohol-related liver disease [6]. However, over the last decades, major improvements in the prevention and treatment of viral hepatitis, such as HBV vaccination, therapy for chronic hepatitis HBV infection, and highly effective oral antivirals for HCV, have been introduced, reducing the burden of chronic viral liver diseases [7].

Conversely, over the past decades, MASLD has emerged as the most prevalent chronic liver disease worldwide, and it is estimated to affect 25% of the general population [8,9]. Its prevalence is projected to increase up to 56% between 2016 and 2030 [10]. MASLD-related obesity and insulin resistance lead to chronic inflammation, altered lipid metabolism, and a pro-carcinogenic state that promotes HCC development [11,12].

These trends in liver disease risk factors are expected to translate into HCC risk in patients with cirrhosis [13,14], but there is little data about the absolute risk of HCC among newer cohorts of patients with cirrhosis from different etiological risk factors.

The aim of this study was to assess the changes in the etiological risk factors for HCC in the recent years in a tertiary university hospital from a country with complete access to antiviral therapies for chronic hepatitis B and C. On top of that, we aimed to analyze the differences in stage at diagnosis and clinical characteristics of HCC patients according to the underlying etiological factor.

## 2. Materials and Methods

### 2.1. Study Population

We performed a retrospective evaluation of all consecutive new cases of HCC diagnosed in adults (≥18 years of age) listed in the Vall d’Hebron Hospital database between January 2019 and December 2022. All clinical records of patients with a diagnosis coded in the hospital database as C22.0 for hepatocellular carcinoma were reviewed, and only those diagnosed as HCC were included. We excluded patients with a previous diagnosis of HCC.

HCC was defined according to the European Association of the Study of the Liver (EASL) criteria, including a histological exam or non-invasive radiological criteria using typical dynamic characteristic appearance (arterial enhancement and delayed washout) on triple-phase CT or MRI [15].

### 2.2. Variables

Demographical and clinical data were collected for those patients. Demographical data included age, gender, and ethnicity. Clinical data included HCC diagnosis date, stage at diagnosis (according to the Barcelona Clinic Liver Cancer (BCLC) staging system [16]), first HCC treatment (no treatment, transplant, resection, ablation, TACE (transarterial chemoembolization), and systemic therapy), presence of cirrhosis at diagnosis, liver disease etiological factor, and comorbidities (arterial hypertension (AH), diabetes, dyslipidemia, obesity, and HIV).

Cirrhosis diagnosis was based on either (1) non-tumoral liver biopsy findings of regenerative nodules surrounded by fibrotic tissue, (2) liver ultrasound showing nodular liver surface, round edge, and hypoechoic nodules in liver parenchyma, (3) transient elastography values over 12.5 kPa, and (4) non-invasive measurements of advanced fibrosis with FIB-4 score (over 3.25) or APRI score (over 1.5) [15].

The etiology of the chronic liver disease and HCC were defined according to the following criteria:

- Chronic hepatitis C, as defined by the presence of anti-HCV antibodies and HCV RNA for more than 6 months. Cured hepatitis C was defined by HCV RNA undetectable either after therapy or spontaneously [17].
- Alcohol-related liver disease (ALD) as a daily ethanol intake higher than 20 g for women and 30 g for men and in the absence of any other etiology [18].
- A combined category of ALD + HCV was established for those patients with both previous etiological factors.
- Chronic hepatitis B, as defined by the presence of hepatitis B surface antigen (HBsAg) for 6 months or more combined with normal or elevated ALT levels [19].
- MASLD, as defined by the presence of either (1) documentation of histological MASLD, (2) physician documentation of MASLD, or (3) obesity, metabolic syndrome, or type 2 diabetes in the absence of any additional etiology [20].
- We combined etiologies with few cases in each group as autoimmune hepatitis, primary biliary cholangitis, hemochromatosis, and Wilson’s disease into a group called others.
- Patients without any of the previous etiological factors were classified as unknown etiology.

### 2.3. Statistical Analysis

For qualitative variables, frequency and percentage were calculated. Categorical variables were compared using the chi-squared test. The results were considered statistically significant when the *p*-value was lower than 0.05. All statistical analyses were carried out using GraphPad Prism 6.

## 3. Results

A total of 352 patients with a new HCC diagnosis between January 2019 and December 2022 were reviewed. The demographical and clinical parameters of these patients are summarized in Table 1. Seventy-eight percent were male with a median age of 71 (20–94) years old. A total of 94.0% percent were Caucasian, 61.6% presented with AH, 37.5% had diabetes, 99.4% had dyslipidemia, 17.3% had obesity, 50.8% were smokers, and 1.4% had anti-HIV antibodies. Eighty-three percent presented with cirrhosis.

At the time of the diagnosis, 28 (8%) patients were BCLC 0, 144 (40.9%) were BCLC A, 58 (16.5%) were BCLC B, 86 (24.4%) were BCLC C, and 36 (10.2%) were BCLC D. A total of 290 (82.4%) from the 352 patients received at least one active treatment for HCC. Regarding curative treatments, 27 (7.7%) patients received an HCC transplant, 44 (12.5%) underwent tumor resection, and 89 (25.3%) underwent ablation. Palliative treatments were indicated in 58 (16.5%) TACE and 72 (20.4%) systemic therapy treatments as first treatments.

**Figure 1:**
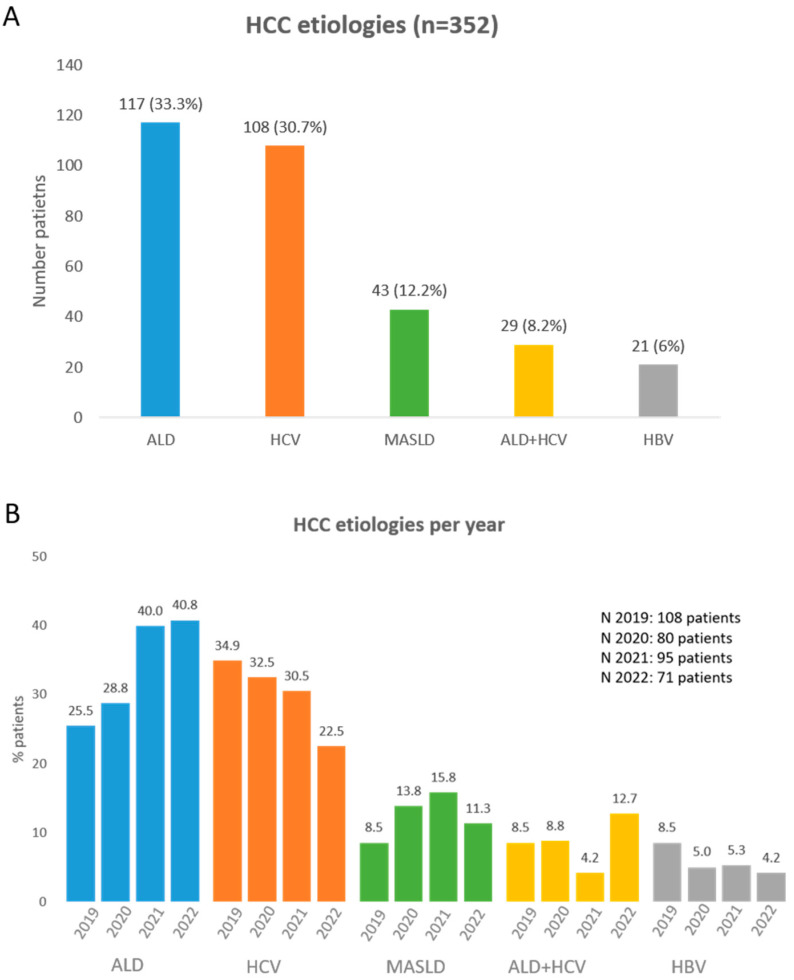
(**A**) Main HCC etiologies between 2019 and 2022 in our center. (**B**) Main HCC etiologies percentage per year.

In our cohort, alcohol related liver disease was the leading etiological risk factor of HCC in 117 (33.3%) of the 352 patients reviewed, followed by HCV in 108 (30.7%) and MASLD in 43 (12.2%) patients. Twenty-nine (8.2%) had a mixed etiological risk factor of ALD and HCV, and twenty-one (6%) patients had HBV (Figure 1A). Ten (2.8%) had other etiological factors, and twenty-four (6.8%) had unknown etiological factors.

A total of 137 patients had HCV infection. Among those, 108 (79%) received oral antivirals and achieved sustained virological response (SVR), and 29 (21%) were untreated.

From the 108 HCV patients who were treated, 87 (81%) were treated with direct-acting antivirals (DAA) and 21 (19%) with interferon +/− ribavirin. The median time between SVR and HCC diagnosis was 5.41 years (range 0.13–27.4 years).

Ninety-six (89%) out of one hundred and eight patients with a resolution of hepatitis C infection had cirrhosis. A similar percentage of cirrhosis, 79% (23 of 29 patients), was found among the patients with active hepatitis C.

Out of the 21 patients with chronic HBV infection, 10 (48%) received antiviral treatment.

Clinical variables such as gender, BCLC stage, the presence of cirrhosis, and median age at the time of diagnosis were reviewed from the patients together with the most common HCC risk factors (Table 1). Gender differences were to be found statistically significant according to etiology (*p* < 0.0001), with the percentage of women being significantly higher (*p* = 0.001) in those with HCV-related HCC compared to those with ALD-related HCC. HBV patients were significantly younger at the time of diagnosis than patients with HCV (*p* = 0.005) and MASLD patients (*p* = 0.001). Significant differences were found as well in the percentage of patients with cirrhosis, it being more frequent in ALD patients diagnosed with HCC than in patients with MASLD or HBV (*p* < 0.05); no significant differences were found in the percentage of patients with cirrhosis with HCV. No significant differences were found in the BCLC stage at time of diagnosis according to etiology; however, when dividing patients with HCV into either cured or active (without HCV treatment), we found that patients with cured HCV were significantly more diagnosed at early stages (BCLC stage 0 or A) (49%) than patients with active HCV (17%) (*p* < 0.05).

Regarding the most frequent etiologies, we observed changes in their frequency with time through the period reviewed. There was an increasing trend in the proportion of patients with ALD and MASLD, while a decline in HBV and HCV was observed. The percentage of patients with alcohol-related liver disease increased from 25.5% to 40.8%, patients with MASLD increased from 8.5% to 11.3%, patients with HCV decreased from 34.9% to 22.5%, and patients with HBV decreased from 8.5% to 4.2% (Figure 1B).

## 4. Discussion

Etiology of liver disease has been reported to be experiencing notable changes. The global MASLD pandemic, policies regarding hepatitis B virus infection vaccination, and accessibility to hepatitis B and C antiviral treatments are changing the burden of related liver disease [21,22]. Whether these epidemiological changes are having an impact already in the specific weight of each one of the underlying liver disease etiologies as a milieu for arising liver cancer has not been extensively reported.

In order to have an insight into the contribution of the different etiological agents and to evaluate their trends in the past few years, in this study, we have reviewed the etiological agents behind all the new diagnosed HCC patients in our center between 2019 and 2022. Reviewing the new 352 HCC cases in this period, we observed that alcohol consumption was the leading cause of HCC in our center, accounting for 33.3% of cases, followed by HCV-related HCC in 30.7% and MASLD in 12.2%.

Different results were seen in some studies performed in the UK and the USA, where the prevalence of viral hepatitis is low, where in the last years, a dramatic increase in the incidence of HCC has been attributed to an increase in MASLD cases [23,24,25,26].

Even though in our center alcohol consumption and HCV infection were the leading causes of HCC, we observed, in the four-year period analyzed, a decreasing trend in the relative weight of HCV infection (from 30.9% to 22.5% of cases) (both in treated and active HCV) and, on the other hand, an increase in ALD- and MASLD-related HCC. ALD almost doubled from 25.5% to 40.8%, and a slight increase was observed in MASLD from 8.5 to 11.3%. This tendency was also observed in a study performed with patients of a French cohort, in which HCV was more frequent, but they observed that MASLD-associated HCC increased from 2.6% to 19.5% between the periods of 1995–1999 and 2010–2014, and HCV-associated HCC decreased from 43.6% to 19.5% [27]. Higher variations in percentages were observed, which were probably due to the longer period of time analyzed.

The rise in ALD-related HCC aligns with the global increase in alcohol intake. Since 1997, the proportion of drinkers has had an annual increase of 0.2%, with it being expected to increase 50% by 2030 [28], suggesting that ALD will continue to be one of the main risk factors of HCC development.

Despite the fact that HCV is decreasing as a cause of chronic liver disease due to the current policies aiming to eradicate HCV infection, from the total patients who had hepatitis C, 79% had been treated for HCV infection, and 89% were cirrhotic. This fact highlights that cured HCV with advanced fibrosis will also continue to be an important contributor to the burden of HCC in the next decade, and it highlights the importance of HCC surveillance not only in cirrhotic patients (F4) but also in treated HCV patients with advanced fibrosis (F3) [29].

In other studies, such as in the ITA.LI.CA study performed in Italy, a significantly lower risk of death has been reported in MASLD-related HCC compared with patients with non-MASLD HCC [30]. No significant differences were found in our cohort, but the percentage of patients who could benefit from curative treatments (BCLC 0 or A) was also higher in those patients with MASLD-related HCC (58%) than in those with non-MASLD HCC (47%).

We observed that HBV patients were younger than those with MASLD and HCV, and they had a lower presence of cirrhosis than patients with ALD, as described before, which is likely due to the intrinsic carcinogenic effect of HBV [31]. Interestingly, a significantly lower number of patients with MASLD also presented with cirrhosis compared to ALD-related HCC patients. Recent studies have also shown that although the highest risk of HCC exists in patients with advanced fibrosis or cirrhosis, 20–50% of HCC cases arise in MASLD patients in the absence of cirrhosis, as seen in our cohort [32,33].

Our results were derived from a unicentric cohort in a limited period of time; however, evolutive changes have been captured, and our data are consistent with recent reports. Multicentric data, ideally from nationwide registry studies, would be the next step to overcome this limitation.

## 5. Conclusions

In conclusion, alcohol-related liver disease and hepatitis C were the main causes of HCC in our center in recent years. In the era of total accessibility to DAA, the development of HCC in HCV-related liver disease patients remains a significant concern, especially in those with advanced fibrosis and cirrhosis. MASLD-related HCC, although growing globally, was not as relevant in our data, accounting for 13% of our patients. Further studies will be necessary to establish MASLD’s influence in other shared risk factors, promoting the progression to an inflammatory state, and triggering carcinogenesis.

## Figures and Tables

**Table 1:** Demographical and clinical characteristics of patients according to different HCC main risk factors.

	Total	ALD	HCV	MASLD	HBV	*p*-Value
Total	352	117	108	43	21	-
Age, median	71	70	74	74.7	60	0.0005
Gender, male	277 (78.7%)	111 (94.9%)	58 (53.7%)	39 (90.7%)	19 (90.5%)	0.0001
Ethnicity						
Caucasian	331 (94%)	116 (99.1%)	102 (94.4%)	40 (93%)	14 (66.7%)	<0.0001
Comorbidities						
AH	217 (61.6%)	77 (65.8%)	64 (59.3%)	36 (83.7%)	9 (42.9%)	0.0056
Diabetes	132 (37.5%)	52 (44.4%)	27 (25%)	31 (72.1%)	5 (23.8%)	<0.0001
Dyslipidemia	350 (99.4%)	38 (32.5%)	15 (13.9%)	23 (53.5%)	7 (33.3%)	<0.0001
Obesity	61 (17.3%)	23 (19.6%)	16 (14.8%)	18 (41.9%)	1 (4.8%)	0.0005
Smoker	179 (50.8%)	81 (69.2%)	41 (38%)	21 (48.8%)	9 (42.9%)	0.0001
Anti-HIV+	5 (1.4%)	0	3 (2.8%)	0	1 (4.8%)	0.1364
Cirrhosis						0.0038
F0-3	61 (17.3%)	7 (6%)	16 (14.8%)	10 (23.3%)	6 (28.6%)	
F4	291 (82.7%)	110 (94%)	92 (85.2%)	33 (76.7%)	15 (71.4%)	
BCLC						0.4991
BCLC 0	28 (8%)	14 (12%)	7 (6.4%)	5 (11.6%)	1 (4.8%)	
BCLC A	144 (40.9%)	55 (47%)	46 (42.2%)	20 (46.5%)	7 (33.3%)	
BCLC B	58 (16.5%)	18 (15.4%)	20 (18.3%)	5 (11.6%)	2 (9.5%)	
BCLC C	86 (24.4%)	18 (15.4%)	29 (26.6%)	11 (25.6%)	8 (38.1%)	
BCLC D	36 (10.2%)	12 (10.2%)	7 (6.4%)	2 (4.7%)	3 (14.3%)	

## Data Availability

The data that support the findings of this study are available from the corresponding author upon reasonable request.
